# Development of sensory neuropathy in streptozotocin-induced diabetic mice

**DOI:** 10.1002/brb3.111

**Published:** 2012-12-23

**Authors:** Tatsufumi Murakami, Takayuki Iwanaga, Yoshinao Ogawa, Yoshiaki Fujita, Eiji Sato, Hironori Yoshitomi, Yoshihide Sunada, Akihiro Nakamura

**Affiliations:** 1 Department of Neurology, Kawasaki Medical SchoolKurashiki, Japan; 2 Department of Clinical Pharmacy, Faculty of Pharmacy and Pharmaceutical Sciences, Fukuyama UniversityFukuyama, Japan; 3 Department of Pharmaceutics, School of Pharmacy, Showa UniversityTokyo, Japan

**Keywords:** Diabetic sensory neuropathy, impaired maturation, sensory conduction velocity, STZ-induced diabetic mice, unmyelinated fiber atrophy

## Abstract

Diabetic polyneuropathy is a major complication of diabetes and the most common cause of peripheral neuropathy. Sensory-dominant neuropathy is the most common type. We previously used streptozotocin (STZ)-induced diabetic ddY mice with sensory neuropathy to evaluate the therapeutic effects of vascular endothelial growth factor and placental growth factor isoforms. In this study, to characterize the development of diabetic sensory neuropathy, electrophysiological, behavioral, and histopathological studies were performed in these diabetic mice. A significant difference in sensory conduction velocity in the tail nerve was observed between healthy and diabetic mice at 1 week after STZ injection. Diabetic mice developed hypoalgesia at 5 weeks after STZ injection. Axon area and myelin thickness of the myelinated fibers were increased in 17-week-old healthy mice compared with those in 8-week-old healthy mice. However, these increases were retarded in 17-week-old diabetic mice. In unmyelinated fibers, axon area was significantly reduced in 17-week-old diabetic mice compared with 8- and 17-week-old healthy mice. These findings suggest that both impaired maturation of myelinated fibers and atrophy of unmyelinated fibers simultaneously occur in the early stage of diabetes in these mice. Our mouse model may be useful for studying the pathogenesis of and therapies for diabetic sensory neuropathy.

## Introduction

Diabetic neuropathy is one of the major complications of diabetes and a frequent cause of peripheral neuropathy. Polyneuropathy is the most common form of diabetic neuropathy and is usually sensory dominant (Llewelyn et al. 2005). Sensory disturbances include paresthesia, pain, or sensory loss in the extremities. Diabetic polyneuropathy is attributed to metabolic and vascular factors including enhanced polyol pathway activity, increased nonenzymatic glycation, oxidative stress, reduced availability of neurotrophic factors, and microvascular insufficiency (Zochodne 2007).

We previously used streptozotocin (STZ)-induced diabetic ddY mice with sensory neuropathy to evaluate the potential therapeutic effects of vascular endothelial growth factor and placental growth factor isoforms (Murakami et al. 2006, 2011). These mice showed increased nociceptive thresholds, that is, hypoalgesia at 6 weeks after STZ injection. Sensory conduction velocity (SCV) in the tail nerve was decreased in these mice at 8 weeks after STZ injection, and a severe reduction in the area showing immunoreactivity for protein gene product 9.5 in epidermal nerves was observed at 9 weeks after STZ injection. Early loss of mechanical sensory and cutaneous axon has also been reported in STZ-induced diabetic C57BL/6 mice (Christianson et al. 2003a,b, 2007). In this study, to characterize the development of diabetic sensory neuropathy, electrophysiological, behavioral, and histopathological studies were performed in STZ-induced diabetic ddY mice. We found that both impaired maturation of myelinated fibers and atrophy of unmyelinated fibers simultaneously occur in the early stage of diabetes in these mice. Our mouse model may be useful for studying the pathogenesis of diabetic polyneuropathy.

## Materials and Methods

### Animal model

Diabetes was induced in 8-week-old male ddY mice (SLC, Shizuoka, Japan) by intraperitoneal injection of STZ (200 mg/kg). The onset of the diabetic state was assessed by the presence of hyperglycemia. The next day or 1 week after the STZ injection, mice with a blood glucose level >16.7 mmol/L were used in experiments. All animal experiments were approved by the Animal Research Committee of Kawasaki Medical School and performed according to the protocols of Kawasaki Medical School.

### Nerve conduction study

All recordings were made with a standard electromyogram (EMG) apparatus (MEB-9402; Nihon Kohden, Tokyo, Japan). Each mouse was anesthetized with 2.5% sevoflurane before recordings. Sensory nerve conduction studies of the tail nerves were performed orthodromically with two pairs of electrodes (Kurokawa et al. 2004). The active stimulating ring electrode was placed 6 cm distal to the active recording needle electrode. Negative peak latency and peak-to-peak amplitude of the sensory nerve action potential (SNAP) were measured (Murakami et al. 2011). The 6-cm distance was divided by the latency, and SCV was calculated. The mice were placed on warm temperature-controlled rubber (ATB-1100; Nihon Kohden). The temperature of the tail was monitored with an electronic thermometer and maintained within 27.0 ± 0.2°C at the active stimulating electrode.

### Paw-pressure test

Mechanical nociceptive thresholds were determined using an Ugo Basile Analgesy Meter (Randall and Selitto 1957). The nociceptive threshold was defined as the force, in grams, at which a mouse struggled to withdraw its hindpaw. The nociceptive threshold was evaluated on the dorsum of the hindpaw bilaterally, and the mean nociceptive thresholds were used for statistical analysis.

### Insulin administration in diabetic mice

Two days after STZ injection, slow-release insulin pellets (Linshin Canada, Inc., Ontario, Canada) were implanted subcutaneously in diabetic mice according to the manufacturer's instruction. The implant dose had enough insulin to last for about 40 days. Blood glucose was measured every week to monitor the efficacy of insulin therapy.

### Morphometric analysis

Because it was difficult to excise nerves from the tails of the mice, sciatic nerves were examined. Segments of mid-thigh sciatic nerves were removed from 8-week-old healthy ddY mice, and from 17-week-old healthy and diabetic ddY mice. Segments were fixed and then embedded in epoxy resin as previously described (Murakami et al. 2006). One-micrometer sections were stained with toluidine blue and observed under a BZ8100 microscope (Keyence, Osaka, Japan). For the morphometric analysis of myelinated fibers, axon area, maximum and minimum axon diameter, axon density, axon number, and myelin area were measured using Dynamic cell count BZ-HIC software (Keyence).

For morphometric analysis of unmyelinated fibers, ultrathin cross-sections of sciatic nerves were obtained using an ultramicrotome, and stained with uranyl acetate and lead citrate. They were examined in a JEM-1400 electron microscope (JEOL, Tokyo, Japan). Randomly selected frames of the sciatic cross-sectional area were obtained. Photographs were enlarged 5000 times and downloaded to the image analysis system. Axon area, maximum and minimum axon diameter, axon density, and axon number were measured using IPLab 3.6.5 software (BD Biosciences, Rockville, MD).

### Statistical analysis

The results are expressed as means ± SEM. Statistical differences between groups with equal standard deviations were verified by either the Student's *t*-test or one-way analysis of variance (ANOVA) followed by Bonferroni's modified *t*-test. The Pearson's correlation coefficient test was also used.

## Results

### Sensory nerve conduction studies of tail nerves

**Figure 1:**
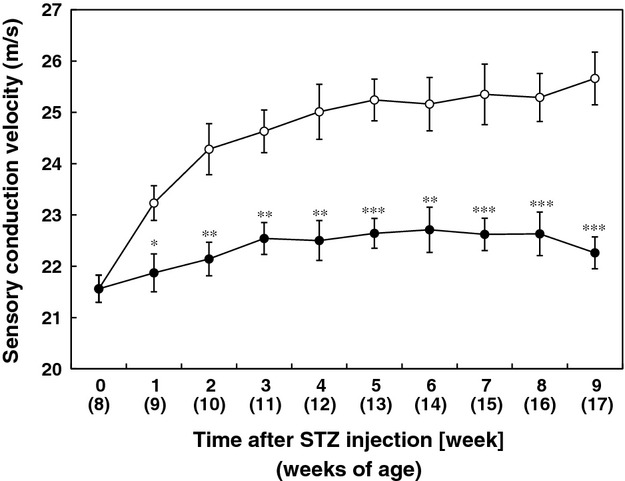
Sensory nerve conduction velocity of tail nerves in healthy and diabetic mice. Diabetic mice (DM) (filled circles) (*n* = 13), healthy mice (control) (open circles) (*n* = 7). DM versus control: *P < 0.05, **P < 0.01, ***P < 0.001, unpaired Student's *t*-test.

Sensory nerve conduction studies of tail nerves were performed weekly from 8 to 17 weeks of age (corresponding to 0–9 weeks after STZ injection) in healthy and diabetic ddY mice. The tail nerve is the longest nerve in the mouse, and tail SCV is probably sensitive to detect neuropathy. The tail nerve could be used repeatedly. SCV and the amplitude of SNAPs were measured. SCV was increased up to 13 weeks of age and then maintained at a steady state until 17 weeks of age in healthy mice (*n* = 7) (Fig. 1). However, a slight increase in SCV was observed in diabetic mice (*n* = 13). The SCV in diabetic mice was significantly lower than that in healthy mice from 1 to 9 weeks after STZ injection. There was no significant difference in the amplitude of SNAPs between groups during the experimental period (data not shown).

### Nociceptive threshold of diabetic and healthy ddY mice

**Figure 2:**
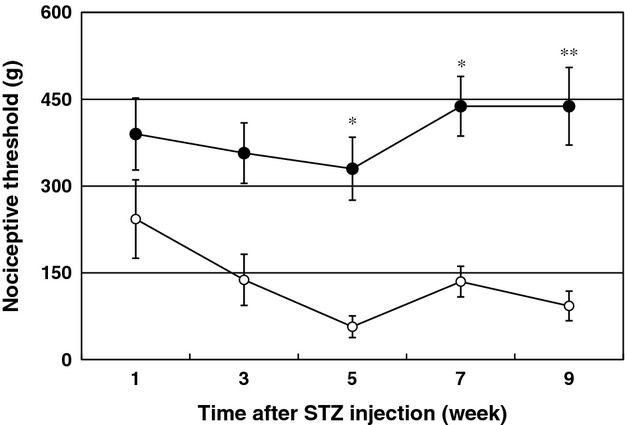
Nociceptive threshold of the hindpaw in healthy and diabetic mice. Diabetic mice (DM) (filled circles) (*n* = 13), healthy ddY mice (control) (open circles) (*n* = 7). DM versus control: *P < 0.05, **P < 0.01, unpaired Student's *t*-test.

The nociceptive threshold of the hindpaw was measured at 1, 3, 5, 7, and 9 weeks after STZ injection in diabetic and healthy ddY mice by the paw-pressure test. The mean nociceptive threshold in diabetic mice (*n* = 13) was higher than that in healthy mice (*n* = 7) throughout the experimental period (Fig. 2). There was a significant difference in the nociceptive threshold between healthy and diabetic mice at 5, 7, and 9 weeks after STZ injection.

**Figure 3:**
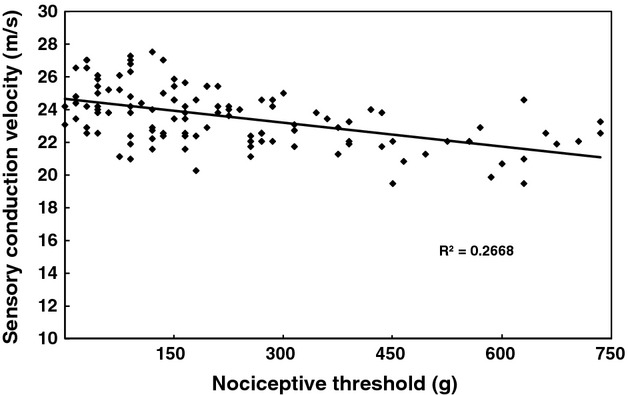
Correlation between sensory conduction velocity (SCV) of the tail nerve and the nociceptive threshold of the hindpaw in healthy and diabetic mice; *n* = 114, *r* = −0.516, *P* < 0.001, Pearson's correlation coefficient test.

The correlation between SCV and the nociceptive threshold was examined in diabetic and healthy mice. We found a significant negative correlation between SCV and the nociceptive threshold (*n* = 114, *r* = −0.516, *P* < 0.001) (Fig. 3).

### SCV and nociceptive threshold of insulin-treated diabetic mice

**Figure 4:**
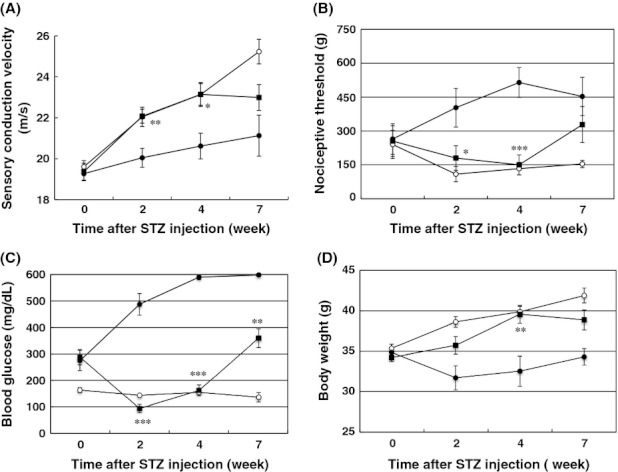
Insulin treatment increased sensory conduction velocity (SCV) of the tail nerve (A) and prevented the elevation of the nociceptive threshold (B) in diabetic mice. Blood glucose levels (C) and body weight (D) were measured at the indicated time points. Nontreated diabetic mice (NT) (filled circles) (*n* = 8), treated diabetic mice (T) (filled squares) (*n* = 8), healthy mice (control) (open circles) (*n* = 8). T versus NT: *P < 0.05, **P < 0.01, ***P < 0.001 (analysis of variance [ANOVA] followed by Bonferroni's modified *t*-test).

Two days after STZ injection, diabetic mice were implanted with insulin pellets (*n* = 8), which remained in place for 7 weeks. Sensory nerve conduction studies of tail nerves and paw-pressure tests were performed at 0, 2, 4, and 7 weeks after STZ injection. We also examined untreated diabetic mice (*n* = 8) and healthy ddY mice (*n* = 8) as control groups. Insulin treatment improved SCV in diabetic mice at 2 and 4 weeks after STZ injection (Fig. 4A), and prevented the elevation of the nociceptive threshold (hypoalgesia) in diabetic mice at 2 and 4 weeks (Fig. 4B). At 7 weeks after STZ injection, blood glucose level was elevated in treated diabetic mice (Fig. 4C), and the preventive effects of the insulin pellets were diminished (Fig. 4A and B).

### Myelinated nerve fibers

We evaluated myelinated fibers of sciatic nerve in the 8-week-old healthy mice and 17-week-old healthy and diabetic ddY mice (Table 1). The axon area of the myelinated nerve was increased in 17-week-old healthy mice (*n* = 6) compared with 8-week-old healthy mice (*n* = 5). In 17-week-old diabetic mice (*n* = 5), the axon area was larger than that in 8-week-old healthy mice, but smaller than that in 17-week-old healthy mice. Maximum and minimum axon diameter showed similar trends to those for axon area. There were no significant differences in axon density or axon number between groups of mice. Myelin area in 17-week-old healthy mice was significantly larger than that in 8-week-old healthy and 17-week-old diabetic mice. There was no significant difference in myelin area between 8-week-old healthy and 17-week-old diabetic mice.

**Table 1:** Morphometric data on myelinated fibers of sciatic nerves

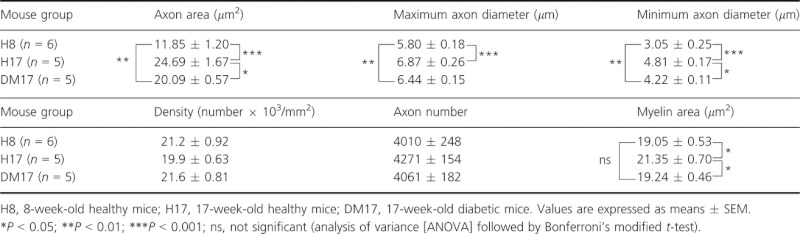

**Figure 5:**
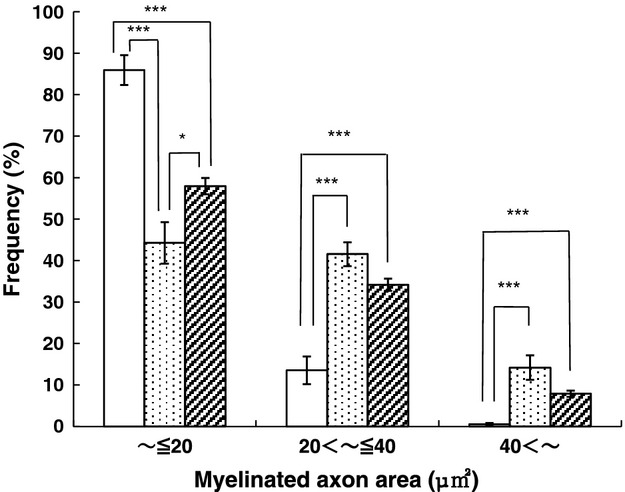
Axon size distribution of myelinated fibers in the sciatic nerves. Eight-week-old healthy mice (H8) (open bars; *n* = 6), 17-week-old healthy mice (H17) (stippled bars; *n* = 5), 17-week-old diabetic mice (DM17) (shaded bars; *n* = 5). H17, DM17 versus H8: ***P < 0.001, H17 versus DM17: *P < 0.05 (analysis of variance [ANOVA] followed by Bonferroni's modified *t*-test).

The axon size distribution of myelinated fibers was examined in each group of mice (Fig. 5). The population of large myelinated axons with a cross-sectional area >40 μm^2^ was significantly increased in 17-week-old healthy and diabetic mice compared with that in 8-week-old healthy mice. The population of small myelinated axons with a cross-sectional area ≤20 μm^2^ was significantly reduced in 17-week-old healthy and diabetic mice compared with that in 8-week-old healthy mice, being more reduced in 17-week-old healthy mice than in 17-week-old diabetic mice.

### Unmyelinated nerve fibers

Unmyelinated fibers of sciatic nerve in each group of mice were also examined under an electron microscope (Table 2). The axon area of unmyelinated fibers was significantly reduced in 17-week-old diabetic mice (*n* = 5) compared with 8-week-old healthy (*n* = 5) and 17-week-old healthy (*n* = 5) mice. There was no significant difference in axon area between 8-week-old and 17-week-old healthy mice, although a slight increase was observed in 17-week-old healthy mice. There was no significant difference in the maximum diameter in each group. The measurements of minimum axon diameters showed similar trends to those for axon area. There were no significant differences in axon density or number between groups of mice.

**Table 2:** Morphometric data on unmyelinated fibers of sciatic nerves

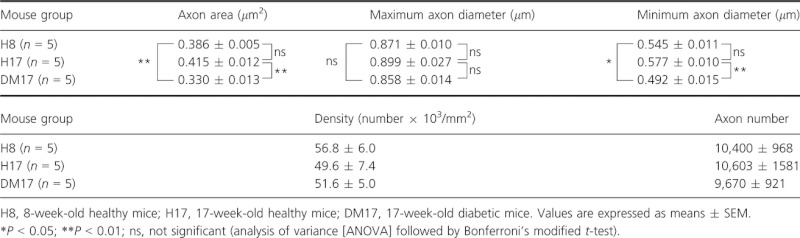

**Figure 6:**
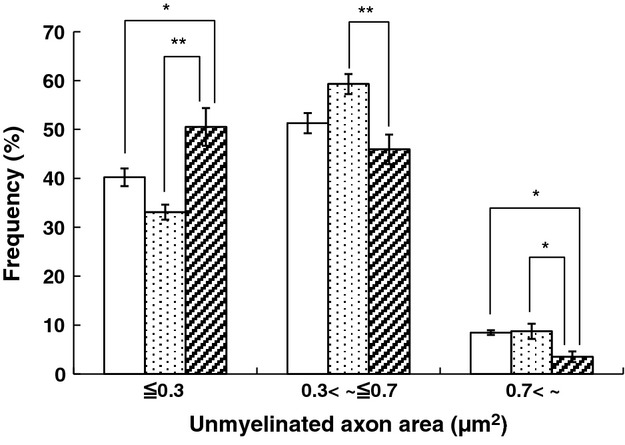
Axon size distribution of unmyelinated fibers in the sciatic nerves. Eight-week-old healthy mice (H8) (open bars; *n* = 5), 17-week-old healthy mice (H17) (stippled bars; *n* = 5), 17-week-old diabetic mice (DM17) (shaded bars; *n* = 5). H8, DM17 versus H17: *P < 0.05, **P < 0.01 (analysis of variance [ANOVA] followed by Bonferroni's modified *t*-test).

The axon size distribution of unmyelinated fibers was examined in each group of mice (Fig. 6). The population of small unmyelinated axons with a cross-sectional area ≤0.3 μm^2^ was significantly increased in 17-week-old diabetic mice compared with that in 8-week-old and 17-week-old healthy mice, while the population of large unmyelinated axons with a cross-sectional area >0.7 μm^2^ was significantly reduced in 17-week-old diabetic mice compared with the other groups of mice.

Electron microscopically, there was no deposition of glycogen within myelinated or unmyelinated axons, which is a characteristic change in diabetic rats (Yagihashi et al. 1990). We could not find axons with degenerative membranous profiles or vacuole formation in axons, as have been previously reported in mutant diabetic mice (Sima and Robertson 1979). There were no structural changes in the endoneural vessels.

## Discussion

It was reported that an early (<1 month of diabetes) motor nerve conduction velocity deficit is not observed in either genetically or STZ-induced diabetic mice (Llewelyn et al. 2005). However, unexpectedly, a significant difference in tail SCV between healthy and diabetic ddY mice was found 1 week after STZ injection in our experiments. Healthy mice showed a progressive increase in tail SCV up to 13 weeks of age, while diabetic mice showed a more gradual increase, suggesting that diabetes might impair the maturation of peripheral nerves.

We also examined the nociceptive thresholds of diabetic ddY mice after STZ injection using the paw-pressure test. Diabetic mice developed hypoalgesia at 5 weeks after STZ injection. A mechanical insensitivity was also reported at 4 weeks after STZ injection in diabetic C57BL/6 and MrgD mice (Johnson et al. 2008), similar to our results. In that study, the numbers of peptidergic intraepidermal nerve fibers were reduced at 4 weeks after STZ injection in diabetic MrgD mice, and this is considered an important process in the loss of sensitivity.

Furthermore, a moderate correlation between the nociceptive threshold and SCV of the tail nerves was identified, suggesting that both myelinated and unmyelinated fibers are simultaneously affected by diabetes. The slowing of conduction and hypoalgesia were not seen in diabetic mice receiving glycemic control with insulin, excluding toxicity of STZ toward the peripheral nerves. Hyperglycemia and insulin deficiency certainly cause sensory neuropathy (Dobretsov et al. 2007)

Next, we histopathologically evaluated the peripheral nerves of 17-week-old healthy and diabetic mice, and compared them with those of 8-week-old healthy mice. In myelinated fibers, axon area and myelin thickness were increased in 17-week-old healthy mice, suggesting that myelinated fiber maturation occurs during this period in these mice. However, their increase was retarded in 17-week-old diabetic mice, consistent with the observations on tail SCV described above. Conduction slowing in diabetic rodents is generally explained by polyol accumulation or axoglial dysfunction (Yagihashi et al. 2001; Llewelyn et al. 2005; Tomlinson and Gardiner 2008). In our diabetic mice, in addition to these factors, peripheral nerve immaturation may be attributed to conduction slowing. In STZ-induced diabetic mice, maturation of regenerated fibers was reported to be impaired after nerve injury (Kennedy and Zochodne 2000). In rats, peripheral maturation rapidly occurs up to 3 months and continues until 9 months (Fraher et al. 1990). Impaired peripheral nerve maturation was noted in STZ-induced diabetic rats (Thomas et al. 1990) and diabetic BB/Wor rats (Kamiya et al. 2009). In the former study, myelinated axon size was reduced in diabetic rats at 9 and 12 months after the onset of diabetes compared with controls.

In contrast to myelinated fibers, the axon area of unmyelinated fibers was significantly reduced in 17-week-old diabetic mice compared with that in 8- and 17-week-old healthy mice, suggesting the existence of unmyelinated fiber atrophy. This finding correlates well with the severe reduction in the area showing immunoreactivity for protein gene product 9.5 in the epidermal nerves of diabetic mice 9 weeks after STZ injection, which we previously reported (Murakami et al. 2011). Although axonal fiber loss was not observed in the sciatic nerve, dying back degeneration had probably begun in the terminals of C-fibers in this mouse model. Our diabetic mice showed earlier and more severe unmyelinated fiber atrophy than other diabetic rodents. In db/db mice, a significant shift of unmyelinated fibers toward a small diameter was recognized at 25 weeks of age (Robertson and Sima 1980). However, STZ-induced diabetic rats did not show a reduction of unmyelinated mean fiber size after 28 weeks of diabetes compared with controls (Yagihashi et al. 1990). In diabetic BB/Wor-rats, unmyelinated fiber sizes and numbers did not change in absolute values between 2 and 10 months, whereas they increased significantly during the same time span in control rats (Kamiya et al. 2009).

In humans, diabetic polyneuropathy is primarily a sensory-dominant neuropathy. Although both large and small fibers are affected by diabetes, small fiber involvement often occurs early (Pittenger and Vinik 2003). Patients with diabetic polyneuropathy usually show positive (paresthesia, allodynia, pain) or negative (numbness, hypoalgesia) sensory symptoms in the extremities (Zochodne 2007). Because unmyelinated fibers were more affected than myelinated ones in our diabetic mice, our mouse model may reflect early diabetic neuropathy in humans. We previously showed that VEGF gene transfer by electroporation improves sensory neuropathy in this diabetic mouse model (Murakami et al. 2006). In addition, the findings of a phase II clinical trial of intramuscular gene transfer using a VEGF plasmid to treat diabetic polyneuropathy have been reported (Kessler 2009; Ropper et al. 2009). Interestingly, sensory loss and neuropathic pain were improved in this trial. Our mouse model may be suitable for screening new drugs to treat diabetic sensory neuropathy (Obrosova 2009).

In summary, we characterized the development of sensory neuropathy in STZ-induced diabetic ddY mice. The slowing of conduction in the tail nerve and hypoalgesia were observed at 1 and 5 weeks after STZ injection, respectively. Both impaired maturation of myelinated fibers and atrophy of unmyelinated fibers simultaneously occurred in the sciatic nerves of these mice. Our mouse model may be useful for studying the pathogenesis of and therapies for diabetic sensory neuropathy.
